# Molecular detection of infectious bronchitis and Newcastle disease viruses in broiler chickens with respiratory signs using Duplex RT-PCR

**Published:** 2014

**Authors:** Aylar Saba Shirvan, Karim Mardani

**Affiliations:** Department of Food Hygiene and Quality Control, Faculty of Veterinary Medicine, Urmia University, Urmia, Iran.

**Keywords:** Duplex-RT-PCR, Infectious bronchitis virus, Newcastle disease virus

## Abstract

Infectious bronchitis (IB) and Newcastle disease (ND) are highly contagious and the most economically important diseases of the poultry affecting respiratory tract and causing economic losses in poultry industry throughout the world. In the present study, the simultaneous detection and differentiation of causative agents of these diseases were investigated using duplex-RT-PCR. RNA was extracted from vaccinal and reference strains of infectious bronchitis virus (IBV) and Newcastle disease virus (NDV) and then cDNA was synthesized. Using two universal primer sets for detection of IBV and NDV, the duplex-RT-PCR was developed. In order to assess the efficiency of the developed duplex RT-PCR, a number of 12 broiler farms with the symptoms of respiratory tract infection was sampled (trachea, lung and kidney were sampled from affected birds suspicious for IBV and NDV infections). After RNA extraction from tissues and cDNA synthesis, the presence of IBV and NDV genome were investigated using duplex-PCR. The results showed that three of twelve examined broiler farms were positive for IBV and two farms were positive for NDV and IBV. The results revealed that the duplex-RT-PCR is a quick and sensitive procedure for simultaneously detecting IBV and NDV in birds with respiratory infections.

## Introduction

Respiratory diseases are among the most devastating diseases in poultry industry because of their major economic losses. In most cases, there are more than one pathogen involving in the pathogenesis of the respiratory diseases.^1^ Among several avian viruses with predilection for the respiratory tract, infectious bronchitis virus (IBV) and Newcastle disease virus (NDV) are the most important viruses of poultry worldwide. Similar respiratory signs of infectious bronchitis (IB) and Newcastle disease (ND) making differential diagnosis of these two diseases difficult.^2^

In broilers, IBV affects weight gain and feed efficiency, and, when complicated with bacterial infections like *E. coli* or *S. aureus*, it causes high mortality and increased condemnations.^3^^-^^5^ IBV, the causative agent of IB is a coronavirus readily undergoes mutation in chickens resulting in the emergence of new variant serotypes and genotypes.^6^ As new strains of IBV emerge, rapid detection of IBV is useful for implementation of control measures, research purposes, and understanding the epidemiology and evolution of IBVs.^7^

Newcastle disease classified as a list A disease by the Office Internationale des Epizooties (OIE), is caused by avian paramyxovirus 1 (APMV-1) or NDV.^8^ The virus is enveloped with a negative-sense, single stranded RNA genome of approximately 15 kb encoding six proteins (nucleoprotein, phosphorprotein, matrix protein, fusion protein, hemagglutinin-neuraminidase protein, and large protein, respectively).^9^

Several laboratory methods such as virus isolation in embryonated eggs and organ cultures and serological tests are available for detecting and differentiating avian viral respiratory infections. However, these methods are time consuming and laborious.^10^^-^^12^ Molecular techniques such as reverse transcription-polymerase chain reaction (RT-PCR), sequencing and real time PCR, have been used for rapid and sensitive detection of IBV and NDV separately.^13^^-^^17^ However, those techniques detect only one specific pathogen at a time. The duplex PCR has the ability to amplify and differentiate multiple specific nucleic acids.^18^ The aim of the present study was to detect and differentiate two common avian viral pathogens using duplex RT-PCR for clinical diagnosis.

## Materials and Methods

**Virus strains. **In this study for developing duplex-PCR, two standard field strains of NDV (Razi institute), a vaccinal strain of IBV named IB88 (Merial, Lyon, France) and one field strain of IBV named 793/B (Faculty of Veterinary Medicine, Tehran, Iran) were used.

**Field samples. **Clinical samples including trachea, liver and kidney were collected from chickens with respiratory symptoms from 12 broiler farms. All farms had a routine history of vaccination against IBV and NDV. Six birds were collected from each broiler farm. Mucosal layer of trachea was scratched by scalpel blade. Tissues of 0.5 cm^3^ was removed from each liver and kidney and then crushed in a sterile mortar with one mL sterile PBS buffer.

**RNA extraction. **Viral RNAs were extracted using RNeasy mini kit (Qiagen, Hilden, Germany) according to the manufacturer's instruction. Extracted viral RNAs were used in subsequent reverse transcription for synthesizing cDNA or stored at –70 ˚C for later use.

**cDNA synthesis and duplex PCR. **cDNA was produced from extracted RNA using reverse transcriptase enzyme. For duplex PCR two sets of universal primers were used in one reaction. The sequence and location of the primers used for amplification of M gene of NDV and 3' UTR of IBV are provided in Table 1. The duplex-RT-PCR was performed in a 25 µL total reaction volume with 0.5 µL of each primer, 2.5 µL 10x PCR Buffer, 1 mM MgCl_2_, 50 µM of each dATP, dCTP, dGTP and dTTP, and 0.5 µM of each primer, 1 U SmarTaq DNA Polymerase, 3 µL cDNA. The thermal profile for duplex-RT-PCR included an initial denaturation in 94 ˚C for 2 min, followed by 35 cycles of 94 ˚C for 45 sec, 55 ˚C for 45 sec, and 72 ˚C for 45 sec, and a final extension cycle at 72 ˚C for 5 min. The duplex-RT-PCR products were separated on 1.5% agarose gel run in 0.5% TBE buffer under constant 80 V for 1 hr and the gel stained by ethidium bromide and visualized under UV light.

## Results

**Fig. 1:**
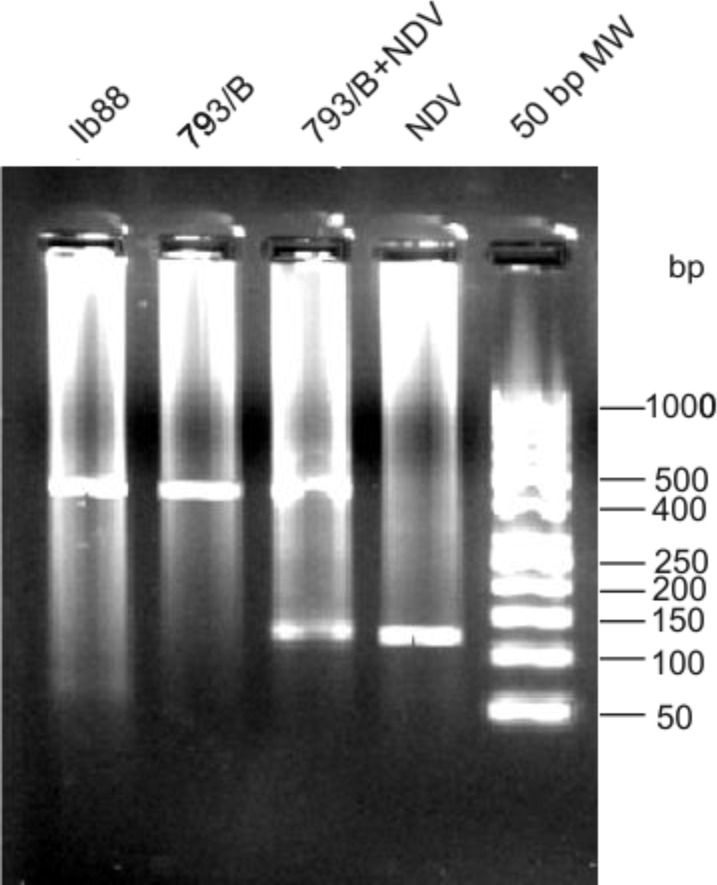
The specificity of RT-PCR for differentiation of IBV and NDV. Lane 1, IB88; Lane 2, 793/B; Lane 3, 793/B+NDV; Lane 4, NDV; Lane 5, 50 bp DNA marker.

**Developing RT-PCR using vaccinal and reference strains of IBV and NDV.** The specificity of duplex-RT-PCR was shown using IB88 and 793/B strains of IBV and two standard strains of NDV. The duplex-RT-PCR products visualize by gel electrophoresis was 433 bp for IBV and 121 bp for NDV (Fig. 1).

**Fig. 2:**
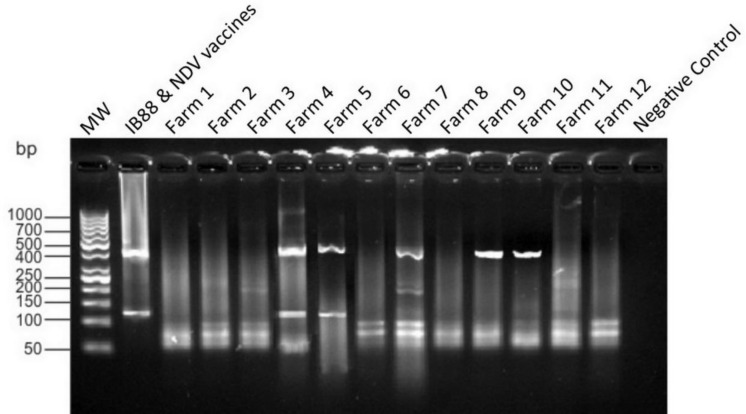
Evaluation of RT-PCR developed in the present study using clinical samples. Lane 1, 50 bp DNA marker. Lane 6 and 7 co-infection with IBV and NDV and lane 9, 11, 12, positive samples for IBV

**Application of developed duplex-RT-PCR for detection and differentiation of IBV and NDV in clinical samples. **The applicability of developed duplex-RT-PCR assay for detection and differentiation of IBV and NDV in the diagnosis was validated examining 12 clinical samples as showed in Fig. 2. Among five positive clinical samples belonged to five different broiler farms, three farms were infected with only one virus and two farms were co-infected with IBV and NDV.

**Table 1:** The sequences and binding sites of primers used in this study

**Primer**	**Primer direction**	**Sequence(5'-3')**	**Gene location**	**Product size (bp)**	**Reference**
**All 1-F**	sense	CAGCGCCAAAACAACAGCG	3' UTR of IBV	433	13
**Del1-R**	Anti-sense	CATTTCCCTGGCGATAGAC	3' UTR of IBV		19
**AMPV1-F**	sense	AGTGATGTGCTCGGACCTTC	M gene of NDV	121	16
**AMPV1-R**	Anti-sense	CCTGAGGAGAGGCATTTGCTA	M gene of NDV		16

## Discussion

Viral respiratory diseases are common causes of economic losses in poultry industry. These diseases cause reduction of growth rate and production, high rate of death, prevention and treatment costs. Quick detection and differentiation of causative viruses can play an important role in controlling these viruses.^20^ IBV and NDV are the viruses that frequently affect the respiratory tract of chickens.^21^ There are several clinically similar viral diseases that can occur in intensive poultry production and require laboratory differential diagnosis. Infectious bronchitis is a global and highly infectious viral disease,^22^ and Newcastle disease is also an economically important viral disease in poultry industry.^23^ Several studies have shown the circulating of different viral respiratory disease including IBV,^24^^-^^27^ NDV^28^ and avian influenza in Iranian poultry farms.^29^^-^^31^

The duplex RT-PCR assay which can be able to quickly identify IBV and NDV will be of great importance in the epidemiology of these viruses especially for controlling of disease transmission among poultry farms and reduction of the economic losses in poultry industries.^32^^, ^^33^ Because of high sensitivity and specificity that PCR offers, since its introduction researchers use it extensively as an indispensable diagnostic method to detect viruses. Using single PCR takes up much time. Therefore using duplex PCR can solve this restriction of PCR.^34^

In the present study, developed duplex RT-PCR was able to detect and differentiate two important viral respiratory diseases of poultry and more importantly the technique was able to simultaneously detect infected birds with both viruses. Since the rapid detection of viral infectious agents in intensive poultry production system is very important, this procedure will be useful to detect more than one infectious agent in the infected farms reducing the time and also costs involved.

Because of the importance of avian respiratory pathogens, many researches have undertaken the detection and differentiation of these pathogens especially AIV, IBV and NDV.^35^^-^^37^ A duplex RT-PCR was developed to detect class І and class ІІ strains of NDV. It was shown that this method had high specificity and high sensitivity.^38^ In another study, Chaharaein *et al.* used duplex RT-PCR for detecting H5, H7 and H9 subtypes of avian influenza viruses.^39^

In the present study two farms were co-infected with IBV and NDV viruses. In was concluded that the developed duplex RT-PCR could be a rapid and economic procedure for detection of IBV and NDV in poultry farms. Using this procedure for detecting these viruses in wild birds is also recommended.

## References

[1] Malik YS, Patnayak DP, Goyal SM (2004). Detection of three avian respiratory viruses by single-tube multiplex reverse transcription-polymerase chain reaction assay. J Vet Diagn Invest.

[2] Ali A, Reynolds DL (2000). A multiplex reverse transcription-polymerase chain reaction assay for Newcastle disease virus and avian pneumovirus (Colorado strain). Avian Dis.

[3] Alvarado IR, Villegas P, El-Attrache J (2003). Evaluation of the protection conferred by commercial vaccines against the California 99 isolate of infectious bronchitis virus. Avian Dis.

[4] Cavanagh D, Gelb J, Cavanagh D, Gelb J (2008). Infectious Bronchitis. Diseases of Poultry.

[5] Van Roekel H, Clarke HK, Bullis KL (1951). Infectious bronchitis. Am J Vet Res.

[6] Dolz R, Pujols J, Ordonez G (2008). Molecular epidemiology and evolution of avian infectious bronchitis virus in Spain over a fourteen-year period. Virology.

[7] De Wit JJ (2000). Detection of infectious bronchitis virus. Avian Pathol.

[8] OIE ( 2012). Newcastle disease. OIE terrestrial manual: manual of diagnostic tests and vaccines for terrestrial animals.

[9] de Leeuw O, Peeters B (1999). Complete nucleotide sequence of Newcastle disease virus: Evidence for the existence of a new genus within the subfamily Paramyxovirinae. J Gen Virol.

[10] Adi AA, Astawa NM, Putra KS (2010). Isolation and Characterization of a Pathogenic Newcastle Disease Virus from a Natural Case in Indonesia. J Vet Med Sci.

[11] Cook JK, Darbyshire JH, Peters RW (1976). The use of chicken tracheal organ cultures for the isolation and assay of avian infectious bronchitis virus. Arch Virol.

[12] Sato T, Sugimori T, Ishii S (1955). Infectious bronchitis of chickens in Japan. I Epidemiological, clinical, and pathological studies and isolation of the causative virus in embryonated eggs. Jpn J Exp Med.

[13] Hewson K, Noormohammadi AH, Devlin JM (2009). Rapid detection and non-subjective characterisation of infectious bronchitis virus isolates using high-resolution melt curve analysis and a mathematical model. Arch Virol.

[14] Meir R, Maharat O, Farnushi Y (2010). Development of a real-time TaqMan® RT-PCR assay for the detection of infectious bronchitis virus in chickens, and comparison of RT-PCR and virus isolation. J Virol Methods.

[15] Chen HW, Wang CH (2010). A Multiplex reverse transcriptase-PCR assay for the genotyping of avian infectious bronchitis viruses. Avian Dis.

[16] Wise MG, Suarez DL, Seal BS (2004). Development of a real-time reverse-transcription PCR for detection of Newcastle disease virus RNA in clinical samples. J Clin Microbiol.

[17] Wang Z, Vreede FT, Mitchell JO (2001). Rapid detection and differentiation of Newcastle disease virus isolates by a triple one-step RT-PCR. Onderstepoort J Vet Res.

[18] Bellau-Pujol S, Vabret A, Legrand L (2005). Development of three multiplex RT-PCR assays for the detection of 12 respiratory RNA viruses. J Virol Methods.

[19] Mardani K, Browning GF, Ignjatovic J (2006). Rapid differentiation of current infectious bronchitis virus vaccine strains and field isolates in Australia. Aust Vet J.

[20] Sakai K, Yada K, Sakabe G (2006). Serological and virological studies of Newcastle disease and avian influenza in slaughter-age ostriches (Struthio camelus) in Japan. J Vet Med Sci.

[21] Villegas P (1998). Viral diseases of the respiratory system. Poult Sci.

[22] Cavanagh D (2007). Coronavirus avian infectious bronchitis virus. Vet Res.

[23] Piacenti AM, King DJ, Seal BS (2006). Pathogenesis of Newcastle disease in commercial and specific pathogen-free turkeys experimentally infected with isolates of different virulence. Vet Pathol.

[24] Akbari Azad G, Vasfi Marandi M, Keyvani Aminae H (2007). Molecular analysis of three Iranian isolates belonged to 793/B serotype of Infectious bronchitis viruses. J Vet Res.

[25] Akbari Azad G, Vasfi Marandi M, Keyvani H (2004). Isolation and molecular identification of infectious bronchitis viruses in poultry farms of Iran. Iran J Vet Med.

[26] Seyfi Abad Shapouri MR, Mayahi M, Assasi K (2004). A survey of the prevalence of infectious bronchitis virus type 4/91 in Iran. Acta Vet Hung.

[27] Seyfi Abad Shoupouri MR, Mayahi M, Charkhkar S (2002). Serotype identification of recent Iranian isolates of infectious bronchitis virus by the type specific multiplex RT-PCR. Arch Razi Instit.

[28] Kianizadeh M, Aini I, Omar AR (2002). Sequence and phylogenetic analysis of the fusion protein cleavage site of Newcastle disease virus field isolates from Iran. Acta Virol.

[29] Fereidouni SR, Werner O, Starick E (2010). Avian influenza virus monitoring in wintering water birds in Iran. 2003-2007. Virol J.

[30] Homayounimehr AR, Dadras H, Shoushtari A (2010). Sequence and phylogenetic analysis of the hemagglutinin genes of H9N2 avian influenza viruses isolated from commercial chickens in Iran. Trop Anim Health Prod.

[31] Toroghi R, Momayez R (2006). Biological and molecular characterization of avian influenza virus (H9N2) isolates from Iran. Acta Virol.

[32] Corbanie EA, Remon JP, Van Reeth K (2007). Spray drying of an attenuated live Newcastle disease vaccine virus intended for respiratory mass vaccination of poultry. Vaccine.

[33] Han GZ, He CQ, Ding NZ (2008). Identification of a natural multi-recombinant of Newcastle disease virus. Virology.

[34] Lee WM, Grindle K, Pappas T (2007). High-throughput, sensitive, and accurate multiplex PCR-microsphere flow cytometry system for large-scale comprehensive detection of respiratory viruses. J Clin Microbiol.

[35] Rashid S, Naeem K, Ahmed Z (2009). Multiplex polymerase chain reaction for the detection and differentiation of avian influenza viruses and other poultry respiratory pathogens. Poult Sci.

[36] Roussan DA, Haddad R, Khawaldeh G (2008). Molecular survey of avian respiratory pathogens in commercial broiler chicken flocks with respiratory diseases in Jordan. Poult Sci.

[37] Tao Q, Wang X, Bao H (2009). Detection and differentiation of four poultry diseases using asymmetric reverse transcription polymerase chain reaction in combination with oligonucleotide micro-arrays. J Vet Diagn Invest.

[38] Liu H, Zhao Y, Zheng D (2011). Multiplex RT-PCR for rapid detection and differentiation of class I and class II Newcastle disease viruses. J Virol Methods.

[39] Chaharaein B, Omar AR, Aini I (2009). Detection of H5, H7 and H9 subtypes of avian influenza viruses by multiplex reverse transcription-polymerase chain reaction. Microbiol Res.

